# Management of Urinoma Formation After Laparoscopic Cryoablation of Renal Cyst

**DOI:** 10.1089/cren.2016.0137

**Published:** 2017-01-01

**Authors:** Shawn X. Li, Lawrence M. Dagrosa, Vernon M. Pais

**Affiliations:** ^1^ Dartmouth-Hitchcock Medical Center, Lebanon, New Hampshire.; ^2^ Dartmouth Geisel School of Medicine, Hanover, New Hampshire.

**Keywords:** urinoma, ureteral stenting, laparoscopic cryoablation, renal cyst

## Abstract

***Aim:*** To describe the presentation and management of a urinoma developing as a complication of laparoscopic cryoablation of a Bosniak III renal cyst.

***Case:*** A 74-year-old woman presented with acute onset of severe left lower abdominal pain 1 day after a laparoscopic cryoablation of a 3 cm multilobular left cystic renal mass. CT revealed a perinephric fluid collection adjacent to the lower pole of the left kidney with active urinary extravasation seen on retrograde pyelogram, confirming the presence of an urinoma. A retrograde ureteral stent was placed with complete resolution of symptoms and the patient was discharged on the first postoperative day. Follow-up CT scans 2 weeks and 2 months after the procedure showed significant reduction of urinoma size, and retrograde pyelogram 5 months after showed resolution of urinoma.

***Conclusion:*** Although often discussed as a possible complication, to our knowledge there are no published case reports in the literature regarding the formation of a urinoma after laparoscopic cryoablation. Furthermore, no data exist on the management of a urinoma after laparoscopic cryoablation. We propose that ureteral stenting is a reasonable approach to the management of this condition.

## Introduction

Urinoma can develop as a result of trauma as well as complications of surgical procedures. Key factors in the development and persistence of a urinoma include initial injury and continued urine production, leading to the extravasation of urine. A case of urinoma development after laparoscopic cryoablation of a renal cyst has not been described in the literature. The differential diagnosis of perirenal fluid collections after laparoscopic cryoablation includes urinoma and other conditions such as seroma, or postoperative abscess. The management of urinoma versus other diagnosis differs and thus it is key to promptly diagnose and treat an urinoma.

## Case

**FIG. 1.**
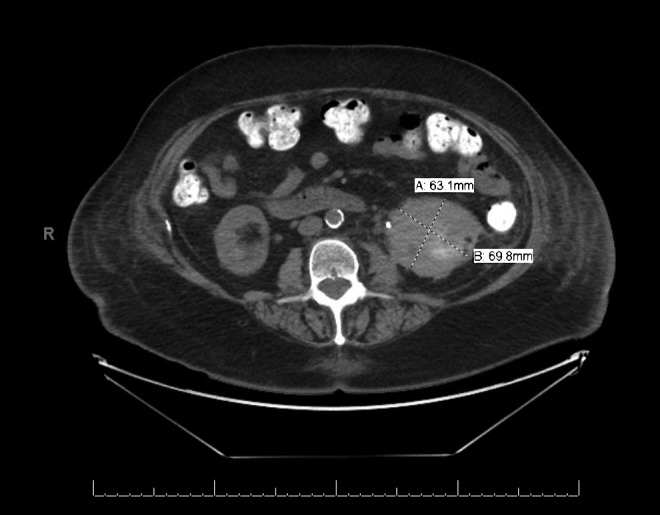
Abdominal CT scan at presentation.

A 74-year-old woman underwent an initially uncomplicated laparoscopic cryoablation for a 3 cm multilobular left Bosniak III renal cyst at an outside institution. She was discharged home on the first postoperative day but represented to the emergency department 24 hours later with sudden and sharp abdominal pain in her lower abdomen. In the emergency department she was found to be afebrile with normal vital signs. She denied hematuria, urgency, dysuria, fevers, or chills. Her urinalysis was normal, she had no leukocytosis, and a basic metabolic panel was within normal limits. Owing to her recent surgery, a delayed phase abdominal CT with contrast was obtained, which revealed an enlarging urinoma emanating from a defect in a lower pole calix (Fig. 1).

Management options considered for the patient included ureteral stenting versus percutaneous drainage. As the patient was not toxic, ureteral stenting was chosen as a less invasive initial intervention.

**FIG. 2.**
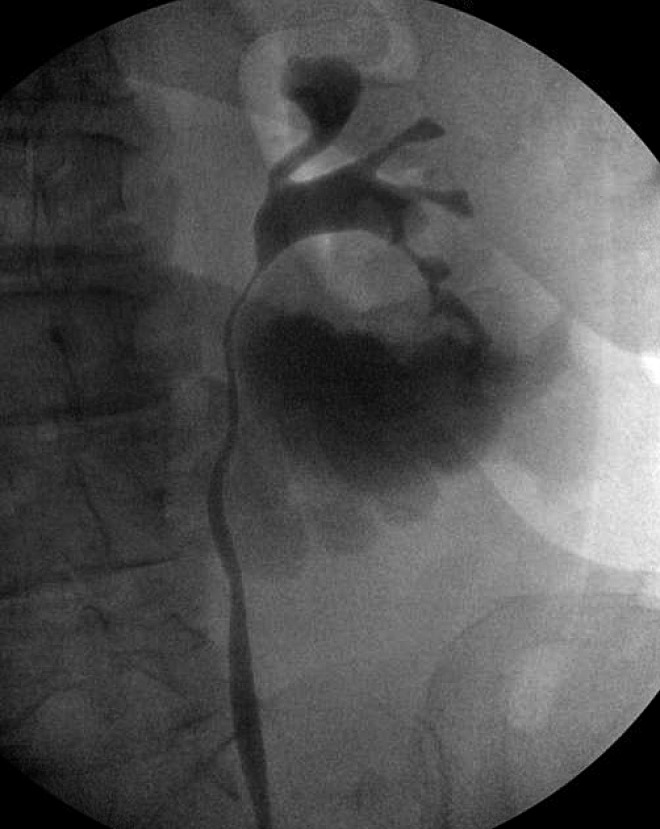
Initial retrograde ureteropyelography showing active extravasation.

**FIG. 3.**
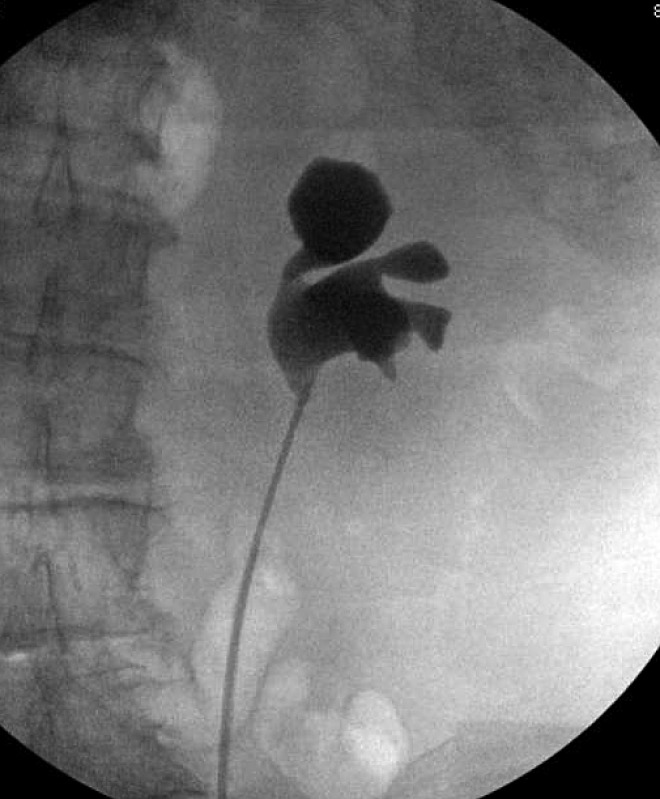
Final retrograde ureteropyelography showing resolution of urine leakage.

A 7F, 26 cm left ureteral stent was placed with intraoperative retrograde ureteropyelography, showing extravasation of contrast from the collecting system into the urinoma (Fig. 2). The day after stent placement, the patient reported complete remission of her abdominal pain. Follow-up CT scan at 2 weeks and 2 months postoperatively showed continued reduction of urinoma size. The patient was ultimately taken to the operating room for retrograde ureteropylography, which did not show urine leak, allowing effective removal of the ureteral stent (Fig. 3).

## Discussion

A drawback to focal cryotherapy of a Bosniak III cyst is that it increases the risk of tract seeding associated with needle biopsies required. Furthermore, complication rate for renal cryoablation was previously described to be 7.7% with urinomas being an uncommon complication. Other possible complications after ablation of renal cysts include hematomas, damage to bowel, ureteral injury, and transient neuropathy. More serious complications of ablative techniques are iatrogenic pneumothoraces as well as fistula formation secondary to hematoma.^1–3^ Puncturing of the collecting system during cryotherapy increases the risk of fistula. In this case, although inadvertent puncture of the collecting system could have occurred, there was no recognized puncture of the collecting system at the time of initial cryoablation.

It is thought that during cryoablation, urinomas arise from thermal injury to the collecting system.^4^ The development and persistence of urinomas can be attributed to three pathophysiologic elements: disruption of the collecting system, continued renal function, and variable distal obstruction.

Urinomas have been described with various incidences with different interventions such as partial nephrectomies (1.6%) and renal cryoablation (0.4%–2.7%).^5,6^ Although urinomas are mechanistically conceivable given the risk for urine leakage, they have not been thoroughly investigated as a complication of laparoscopic cryoablation of renal cysts—most likely because of the rarity of the condition as well as the possibility of delayed presentation as it is with this case.^7^

If urinomas form, they are best detected through delayed phase CT scan. Ultrasound is also a possible image modality but it cannot detect the difference between urinoma, seroma, or abscess.^7^ In addition to identifying the urinoma, the source of the leak can also be observed through retrograde ureterogram.

Treatment of urinomas ought to be focused on diverting urine away from the point of injury. In the case of blunt renal trauma, ureteral stenting and percutaneous drainage have been demonstrated as effective treatments. Umbreit et al.^8^ reported that 17% of patients developed symptomatic urinomas after blunt renal trauma. A total of 81% (13/16) of urinomas resolved with either ureteral stenting or percutaneous drainage. Seven patients underwent percutaneous drainage, but three out of the seven patients needed subsequent ureteral stenting.^8^

Similar to blunt trauma, laparoscopic cryoablation of renal cysts is also a potential cause of urinoma. However, with cryoablation, the damaged area is often smaller and more localized. In addition, cryoablation does not require intraoperative ischemia, thus has a slightly lower risk of parenchymal injury.^9^ Thus, the management of urinoma after laparoscopic cryoablation should be similar to that of blunt trauma, but conceivably more conservative.

One can argue that the more efficacious of the two (ureteral stenting versus percutaneous drainage) should be used first to reduce number of repeat procedures. However, there are no randomized prospective studies directly comparing percutaneous drainage and ureteral stenting. From a patient comfort perspective, ureteral stenting has many advantages. Besides being the less invasive of the two procedures, ureteral stenting also eliminates the risk of dislodging a nephrostomy tube. In addition, internal drainage is more cosmetically appealing and less of a social burden. Thus in the situation of mild symptoms in a nontoxic patient, urinary stenting is a reasonable first intervention.

## Conclusion

Although frequently discussed as a possible complication, we add to the literature a case report of a urinoma after a laparoscopic cryoablation of a renal cyst. This patient was managed conservatively with retrograde ureteral stent placement and serial imaging.
